# Characterization of Grain Structure Using Micro-CT and Identification of Related Candidate Genes by QTL Mapping in Foxtail Millet

**DOI:** 10.3390/plants14233603

**Published:** 2025-11-26

**Authors:** Meixia Tan, Yang Yang, Jiarong Zhang, Dake Guo, Biao Lei, Yuyuan Miao, Fangfang Ma, Siyu Hou, Jiwan Han, Xiaodong Liu, Yuanhuai Han

**Affiliations:** 1 College of Agriculture, Shanxi Agricultural University, Jinzhong 030810, China; tanmeixia186@163.com (M.T.); yang116@sxau.edu.cn (Y.Y.); 18811137601@163.com (J.Z.); 18434764349@163.com (D.G.); lb19980129@163.com (B.L.); mff1984abc@163.com (F.M.); 18635068055@163.com (S.H.); 2 College of Software, Shanxi Agricultural University, Jinzhong 030810, China; 16634257266@163.com (Y.M.); hanjiwan@gmail.com (J.H.)

**Keywords:** *Setaria italica*, grain structure, Micro-CT scanning, QTL mapping, transcriptome analysis

## Abstract

Foxtail millet is a plant that is highly drought-resistant and rich in nutrients. Its grain structure traits are linked with water uptake during grain germination, which is closely related to plant density for high yield under rain fed regions; however, there is no related research. Using Micro-CT technology, we investigated a total of 15 internal structure traits of foxtail millet grains, such as the volume and surface area of the embryo, endosperm, cavity, hull, and whole grain, and mapped relevant quantitative trait loci (QTLs) using recombination inbred lines (RILs). With phenotypic variations in these traits and genome sequences of 100 foxtail millet RILs, four QTLs were identified. In combination with transcriptome profiling during grain development, we identified seven candidate genes that may play a role in the regulation of grain structure in foxtail millet.

## 1. Introduction

Given the global decrease in water resources and environmental deterioration, foxtail millet has attracted worldwide attention because of its outstanding characteristics such as drought tolerance, water saving, abundant nutrition, and environmental adaptability [1,2,3,4]. Due to its small grain size with a thousand-grain weight about 3 g, the seed of foxtail millet is sowed just 3 cm deep into the soil to facilitate the emergence of seedlings. Rain-fed foxtail millet relies on soil moisture in order for the grains to germinate successfully. The ideal timing for germination is before the top layer of soil dries out. The hull and caryopses of the grains are crucial for the uptake of water from the soil, which is essential for timely germination and achieving the desired plant density necessary for a good yield [5,6,7]. However, there have been limited studies on this research area in foxtail millet, which restricts the discovery of relevant genes for molecular breeding for drought tolerance varieties with quick germination.

To study seed structure, traditionally, the grains were sectioned and observed using an optical microscope [8,9]. Not only is it difficult to obtain the comprehensive grain structure in detail, but also time-consuming and laborious, making it prone to errors. Although fluorescence microscopy enables high-resolution imaging of cellular and subcellular structures through fluorescent dye labeling, it presents challenges when imaging deep tissues [10,11,12,13]. While the scanning electron microscope (SEM) can produce high-resolution images, it requires spraying metals on the sample surface and is limited to capturing the image of the sample surface only. The transmission electron microscope (TEM) offers a high resolution; however, it is also limited to generating simple two-dimensional structure images and the sample preparation is long and laborious [14,15,16,17].

Micro-CT utilizes differences in X-ray absorption and transmittance of plant tissues to reveal their three-dimensional structure through 2D or 3D images [18]. It has been used to study the 3D structures of maize kernels [19], American field pansy (*Viola bicolor*) seeds [20], barley flowers [21], *Arabidopsis* flowers [21], and leaves of a range of species (including both dicots and monocots) [22]. So far, Micro-CT has succeeded in crop phenomics analysis, but only a limited number of studies linking phenomic data with genotype data have been reported.

The grain of the grass family (*Poaceae*) represents a highly specialized fruit type that has been crucial to human civilization, forming the nutritional foundation of major cereals such as foxtail millet (*Setaria italica*), rice (*Oryza sativa*), sorghum (*Sorghum bicolor*), and maize (*Zea mays*). The grain has a complex structure derived from a floret with a caryopsis that is encased by the hull [23]. The caryopsis is a dry, indehiscent fruit characterized by the fusion of the exocarp, mesocarp, and endocarp, with the seed coat into a thin, hard layer that is sometimes called caryopsis coat. Additionally, in some species, the caryopsis is covered by a structure called the hull, which is not fused to the caryopsis, but adheres tightly to it and can be difficult to remove. The hull is produced by the transformation of the bract(s) that are subtending the flower. These bracts are called the lemma and palea. The grain of *Setaria* sp. is caryopsis with hull [24]. The caryopsis consists of three genetically distinct components: (i) the diploid maternal tissues including the pericarp, testa, and remnants of the nucellus; (ii) the triploid filial endosperm tissue; and (iii) the diploid filial embryo tissue [9]. This anatomical structure exhibits remarkable evolutionary adaptations for protection, dormancy, and efficient germination [25,26].

Quantitative trait loci (QTL) analysis is a rapid and accurate method for identifying the major genes that control quantitative traits. An F2 population consisting of 167 individuals derived from Yugu1 × Longgu7 was used to detect QTLs associated with agronomic and yield trait in foxtail millet, resulting in the identification of 29 QTLs linked with 11 traits [27]. These QTLs related to hull color traits were detected using an RIL population, and five candidate genes were identified [28]. The integrative quantitative trait mapping and transcriptome analysis is an effective strategy to narrow down the mapping area or reduce the number of candidate genes. Using this approach, a number of candidate genes are identified in different crops such as genes related to seed size in einkorn wheat (*Triticum monococcum* ssp. *monococcum*) and pericarp thickness in sweetcorn [29,30].

In this study, 3D images of foxtail millet grains were obtained using Micro-CT technology, and 15 traits data such as volume and surface area of embryo, endosperm, cavity, hull, and whole grain were analyzed. Through the analysis of 15 traits in 100 RILs, the candidate genes were identified using an integrative analysis of QTL mapping and transcriptome. These results not only lay the foundation for the study of the regulatory mechanism of related traits in the structure of foxtail millet grains, but also provide a basis for research on improving the water absorption rate of foxtail millet grains during germination.

## 2. Results

### 2.1. Three-Dimensional Structure of All Tested Grains

**Figure 1:**
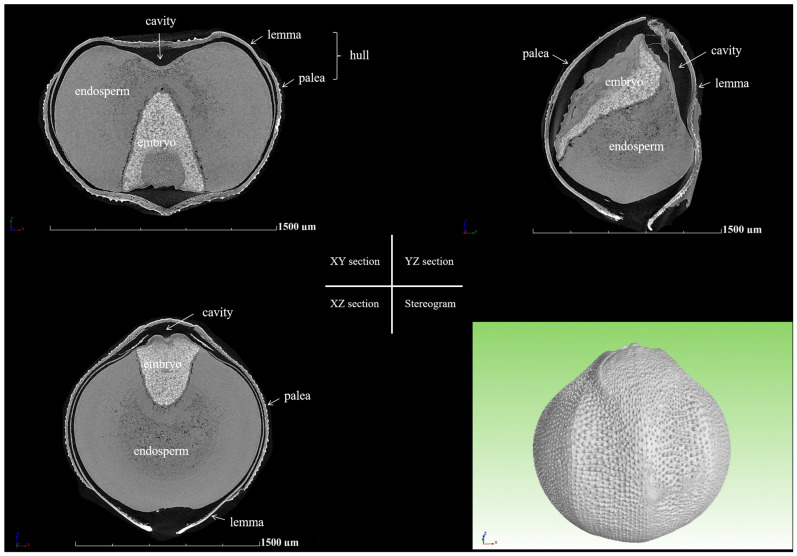
Scanning images of a foxtail millet grain (germplasm GBS) with botanical terms [24]. In this paper, we define the term “cavity” as an expression for the gap between caryopsis and hull. The caryopsis consists of the embryo, endosperm, and caryopsis coat (the caryopsis coat, which is thin layers of dead cells closely adhering the endosperm and embryo, is relatively poorly contrasted in the Micro-CT image). The caryopsis is covered by a structure called the hull, which can be divided into the lemma and palea.

**Figure 2:**
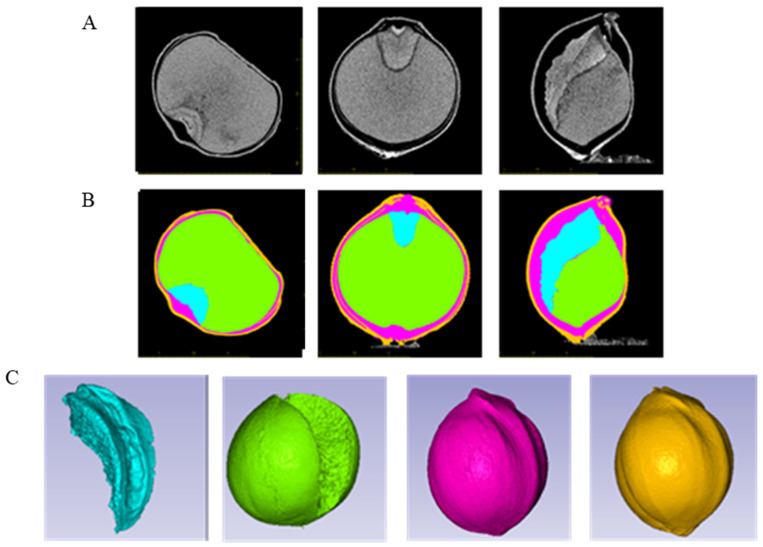
Visualization of different portions of a mature foxtail millet grain after being labeled and segmented (germplasm GBS). (**A**) Micro-CT reconstruction images in different cross-sectional directions. (**B**) Different tissues were labeled different colors. (**C**) The embryo (blue), endosperm (green), cavity (purple), and hull (yellow) were segmented, respectively.

Based on high-resolution Micro-CT reconstruction images, foxtail millet grains are composed of four parts, embryo, endosperm, cavity, and hull (Figure 1). The different parts exhibit varying X-ray absorption, resulting in varying grayscale levels. To accurately identify and separate these parts, region growing techniques were employed. The embryo, endosperm, cavity, and hull were, respectively, assigned the colors blue, green, purple, and yellow (Figure 2B). Finally, by manually adjusting the threshold values for the regions with small differences that are difficult to distinguish, precise segmentation of the embryo, endosperm, cavity, and hull was achieved (Figure 2C), and the 3D volume, surface area, and hull thickness were calculated using the AVIZO 8.0 software.

### 2.2. Relationship Between Different Traits of All Tested Grains

**Figure 3:**
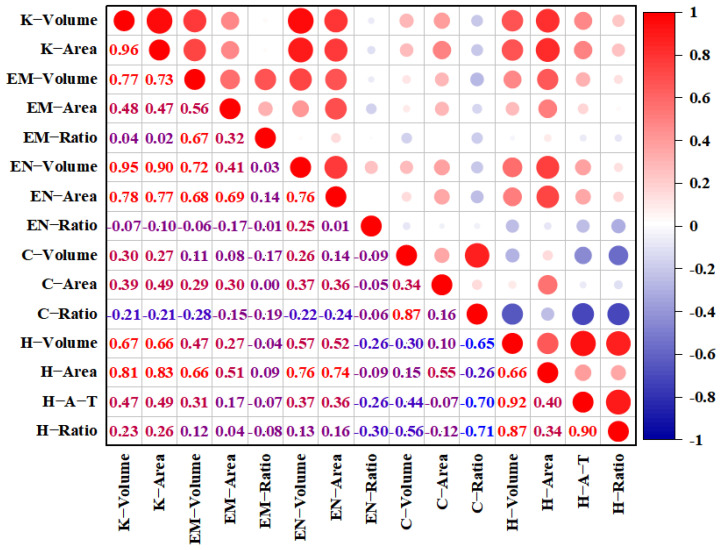
Correlation analyses of grain phenotypic traits. Note: The corresponding Pearson’s correlation coefficients are displayed in the lower left panels. The intensity of the color of the numbers represents the strength of the correlation. Red numbers indicate a positive correlation; blue numbers indicate a negative correlation. In the upper right panels, red and blue denote positive and negative correlations, respectively. K, the whole grain; EM, embryo; EN, endosperm; C, cavity; H, hull; H-A-T, the average thickness of the hull.

Correlation analysis of grain phenotypic traits revealed significant positive correlations among the volume of the embryo (EM-Volume), the volume of the endosperm (EN-Volume), the volume of the hull (H-Volume), and the volume of the grain (K-Volume), with correlation coefficients of 0.77, 0.95, and 0.67, respectively (Figure 3). H-Volume exhibited positive correlations with the average thickness of the hull (H-A-T), the proportion of hull volume (H-Ratio), and the surface area of the hull (H-Area) with the correlation coefficients of 0.92, 0.87, and 0.66, respectively. The correlation coefficient between H-Ratio and H-A-T was 0.90. The proportion of cavity volume (C-Ratio) showed negative correlations with H-Volume, H-A-T, and H-Ratio, with correlation coefficients of −0.65, −0.70, and −0.71, respectively. C-Ratio exhibited weak negative correlations with the other ten traits.

### 2.3. Phenotypic Analysis for Foxtail Millet Grains

The grain phenotypic data of the two parents, JG21 and GBS, were collected using Micro-CT. As shown in Table 1, the volume and area of the embryo, endosperm, hull, and whole grain of JG21 were greater compared to GBS. Statistical analysis revealed substantial phenotypic variations in the grain phenotypic traits of the RIL population. The coefficient of phenotypic variation (CV) ranged from 6.71% to 20.57%. Using the online software SPSSAU (https://spssau.com/indexs.html, accessed on 25 July 2025) and the Shapiro–Wilk test method, it was found that only four traits, namely K-Volume, the surface area of the embryo (EM-Area), the surface area of the endosperm (EN-Area), and the proportion of the endosperm volume (EN-Ratio), showed significance (*p* < 0.05). However, for all traits, the absolute value of skewness was less than 3, and the absolute value of kurtosis was less than 10. This indicates that all traits exhibit a normal distribution.

### 2.4. Identification of QTLs Related to Grain Structures in Foxtail Millet

**Figure 4:**
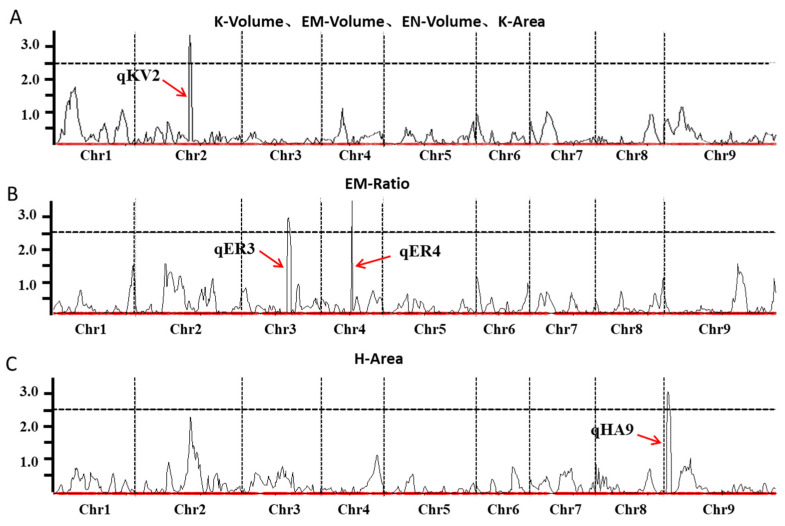
QTLs localization of grain phenotypic traits. (**A**) QTL localization of grain volume, embryo volume and endosperm volume; (**B**) QTLs localization of the proportion of embryo volume; (**C**) QTL localization of hull surface area. Note: The *x*-axis represents the chromosome and the *y*-axis represents the LOD value. The black horizontal dotted line is the significance threshold of the LOD value, and the black vertical dotted line is the chromosome discrimination line. qKV2, qER3, qER4, and qHA9 are the names of the loci of quantitative traits. K, the whole grain; EM, embryo; EN, endosperm; H, hull.

A total of four QTLs were identified for the six traits (Figure 4 and Table 2). One QTL was found on chromosome 2, explaining 14.51~17.09% of the phenotypic variation (PVE) for K-Volume, EM-Volume, EN-Volume, and K-Area, with logarithm of odds scores (LOD) ranging from 2.66 to 3.38. The additive effects (Add) were positive. For EM-Ratio, two QTLs were detected on chromosomes 3 and 4, explaining 11.40% and 12.23% of the PVE, with LOD scores of 2.95 and 3.49, respectively. The Add values were opposite for these QTLs. Additionally, one QTL was identified on chromosome 9 for H-Area, explaining 13.79% of the PVE, with a LOD score of 3.05. The Add value for this QTL was negative.

### 2.5. Differentially Expressed Genes During Grain Development Between Varieties with Different Grain Structures

**Figure 5:**
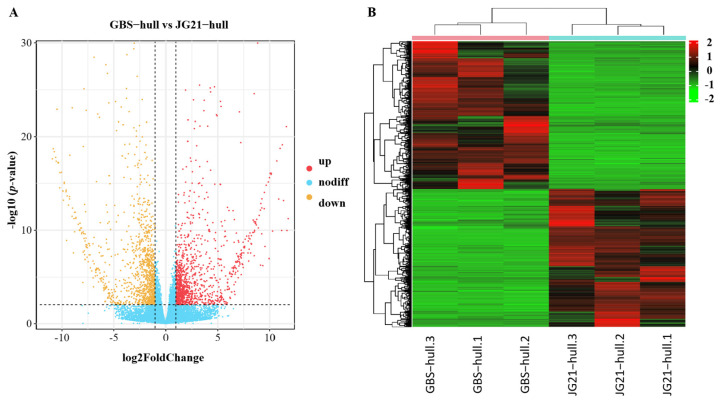

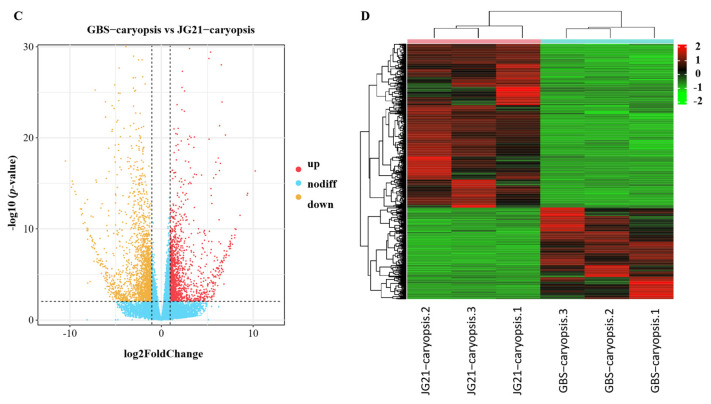
The differentially expressed genes (DEGs) identified between GBS and JG21. (**A**) Volcano plot of DEGs identified in the hull between GBS and JG21. (**B**) Hierarchical clustering heat map of DEGs identified in the hull between GBS and JG21. (**C**) Volcano plot of DEGs identified in the caryopsis between GBS and JG21. (**D**) Hierarchical clustering heat map of DEGs identified in the caryopsis between GBS and JG21. Note: In the volcano plot, the *x*-axis represents the fold change (log2FoldChange). A larger absolute value on the *x*-axis indicates a greater fold change in expression between GBS and JG21; the *y*-axis indicates the significance level of expression differences. Upregulated genes are represented by red dots, downregulated genes by yellow dots, and genes with no significant changes by blue dots. In the cluster analysis heatmap, the *x*-axis represents samples and the *y*-axis represents differentially expressed genes. The left side clusters genes based on expression similarity, and the top clusters each sample based on expression profile similarity. Expression levels increase gradually from green to red, with numbers indicating the normalized relative expression levels.

To further identify the candidate genes of grain structural traits, we performed a transcriptome analysis on the caryopsis and hull of two varieties with significantly different grain structures during the mid-grain-filling stage. This resulted in the generation of approximately 88.20 Gb of total nucleotide data from GBS and JG21 through RNA sequencing. We identified a total of 3045 differentially expressed genes (DEGs) in the hull between GBS and JG21 (|log2FoldChange| ≥ 1 and *p* values < 0.05). Among these genes, 1454 were upregulated and 1591 were downregulated in JG21 (Figure 5A,B and Table S1). We also identified a total of 4312 DEGs in the caryopsis between GBS and JG21 (|log2FoldChange| ≥ 1 and *p* values < 0.05). Among these genes, 2439 were upregulated and 1873 were downregulated in JG21 (Figure 5C,D and Table S2).

### 2.6. Expression Analysis of Candidate Genes Related to Grain Structure

**Figure 6:**
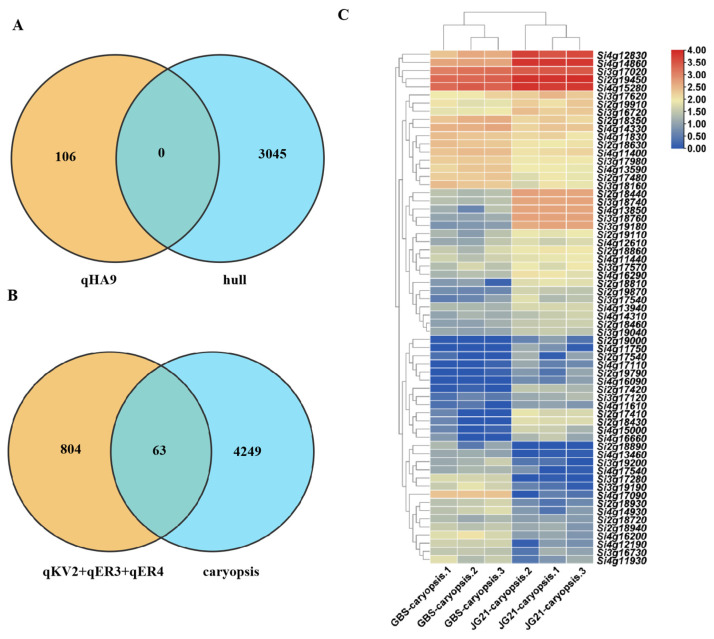
Expression analysis of genes within QTL localization intervals. (**A**) Expression analysis of genes within the qHA9 interval. (**B**) Expression analysis of genes within the qKV2, qER3, and qER4 intervals. (**C**) Heatmap of genes expression analysis within the qKV2, qER3, and qER4 intervals. Note: In the heatmap, the *x*-axis represents samples and the *y*-axis represents differentially expressed genes. On the left side, genes are clustered based on expression similarity, and at the top, each sample is clustered based on the similarity of their expression profiles. The expression levels gradually increase from blue to red, with numbers indicating the normalized relative expression levels.

Genes located within the qHA9 interval did not exhibit differential expression in the hulls between GBS and JG21. Within the qKV2, qER3, and qER4 intervals, a total of 63 genes showed differential expression in the caryopsis between GBS and JG21, among which *Si2g18440*, *Si3g18740*, *Si3g18760*, *Si3g19180*, *Si4g12830*, *Si4g14860*, and *Si4g17090* displayed significant differences (Figure 6).

### 2.7. Functional Prediction of Candidate Genes for Grain Structure

A total of seven candidate genes were found to be displaying significant differences within the qKV2, qER3, and qER4 intervals. Their homologous genes in *Oryza sativa* (L.) were found in the Phytozome 13 database, and the functional annotation information of homologous genes was searched in the China Rice Data Center to preliminarily predict the functions of the candidate genes. According to the annotation of homologous genes, the seven candidate genes were named *SiUDP82A1*, *SiUDP*, *SiUDP73E1*, *SiWAT1*, *SiLEA1*, *SiLEA31*, and *SiAP2* (See Table 3).

## 3. Discussion

Foxtail millet grain structural characteristics play an important role in germination as a rain-fed crop. However, foxtail millet has small grain size and a complex and irregular structure, which makes it difficult to study. Micro-CT technology overcomes the limitations of traditional phenotypic identification and analysis by providing non-destructive three-dimensional structural information of plant tissues [18,21]. This technology opens up opportunities for combined phenotypic and genotypic studies of small grains such as those of foxtail millet. Grain structural traits are quantitative traits controlled by multiple genes [31,36]. We used QTL mapping in combination with transcriptome analysis to identify the candidate genes regulating the traits related to grain structures in foxtail millet.

### 3.1. Grain Structure Characteristics

Compared to photon- and electron-based tomography, Micro-CT combines the advantages of high resolution and excellent deep penetration. Significant differences in morphology and internal structure were observed with Micro-CT technique among different types of corn grains, and there were also significant correlations among different traits of corn grains [19]. Similarly, using Micro-CT technique we investigated the grain structure characteristics of foxtail millet, and significant differences in grain structure were found among different varieties. There were significant correlations among different traits of foxtail millet, for example, the volume of embryo, endosperm, and husk is significantly positively correlated with the volume of the whole grain. Moreover, we observed noteworthy correlations among different traits of foxtail millet grain. Notably, there was a strong positive correlation between the volumes of the embryo, endosperm, and hull with the whole grain volume.

However, Micro-CT also presents certain limitations, including high cost and a resolution that remains amenable to further enhancement. Specifically, the caryopsis coat—which is thin layers of dead cells closely adhering the endosperm and embryo—is relatively poorly contrasted in the Micro-CT image. Additionally, the degree of rugosity of the lemma, an important character in taxonomic diagnosis, is also imaged with limited contrast, thereby constraining the diagnostic utility of the Micro-CT [25,26].

### 3.2. QTL Mapping and Identification of Candidate Genes in Combination with Transcriptome Profiling

Quantitative trait loci (QTL) mapping is increasingly important in molecular breeding, including marker-assisted selection (MAS) and gene discovery. In recent years, researchers have utilized germplasm materials with various genetic backgrounds to create populations for QTL mapping of important agronomic traits in foxtail millet [27,28]. In this study, we conducted QTL mapping of grain structure traits using 100 lines of a RIL population and identified a total of four QTLs for six traits. The whole grain volume, embryo volume, endosperm volume, and whole grain area were all located within the same genomic region. Integrating quantitative traits and transcriptome analysis can effectively narrow down the localization region or reduce the number of candidate genes [29,30]. In this study, QTL mapping combined with transcriptome analysis revealed 63 genes showing differential expression within the QTL interval in parent grains. Subsequently, the nucleic acid and amino acid sequences of these 63 genes were compared and searched in multiple databases, resulting in the identification of 7 genes that are associated with grain size (Table 3).

Studies in rice and *Arabidopsis* have demonstrated that proteins participating in signaling pathways mediated by proteasomal degradation, phytohormones, and G proteins regulate seed size by influencing cell proliferation and cell elongation [37]. Ethylene-responsive transcription factors play a significant regulatory role in various biological and physiological processes, such as plant morphogenesis, stress response mechanisms, hormone signaling, and metabolite regulation. In rice, the APETALA2/ethylene-responsive element binding protein family transcription factor, *rsr1* mutants, exhibit larger grain size, increased grain mass, and therefore higher yield [35]. In this study, the candidate gene *SiAP2* was annotated as an ethylene-responsive transcription factor. Its expression level in the caryopsis of JG21 was significantly lower than that of GBS, whereas the grain volume of JG21 was larger than that of GBS, indicating that this transcription factor may negatively regulate grain size.

Auxin plays a crucial role in plant cell elongation and division. *Arabidopsis WALLS ARE THIN1* (*WAT1*) facilitates auxin export from vacuoles and is required for auxin homeostasis [33]. In this study, the candidate gene *SiWAT1* was identified as the WAT1-related protein *At5g07050*. Its expression level in the caryopsis of JG21 was higher than that of GBS, suggesting a potential role in auxin transport within the grains, thereby contributing to increased grain size.

Flavonoids directly influence auxin transporter, distribution, activity, and content, thereby regulating polar auxin transport. UDP-glycosyltransferases regulate flavonoid activity and stability. In rice, *GSA1* encodes a UDP-glucosyltransferase that exhibits glucosyltransferase activity toward flavonoids. *GSA1* regulates grain size by modulating cell proliferation and expansion [31]. Natural variations in *EDR1* (*Endosperm Development in Rice*) significantly reduced UDP-glucosyltransferase activity, resulting in abnormal endosperm development in the paddy rice. *EDR1* and *GSA1* might have similar functions [32]. In our study, the candidate genes *SiUDP82A1* and *SiUDP73E1* were predicted to encode UDP-glycosyltransferases, while *SiUDP* was predicted to encode a UDP-glucosyl transferase. The expression level of *SiUDP82A1* in the caryopsis of JG21 was significantly higher than that of GBS, indicating that it may serve as a major gene involved in the regulation of caryopsis size. On the other hand, the expression levels of *SiUDP* and *SiUDP73E1* in the caryopsis of JG21 were slightly higher than in GBS, suggesting they may act as minor genes involved in regulating grain size.

*Arabidopsis* mutant *mdn1-1* exhibits an increased seed size and over-accumulation of LEA (Late Embryogenesis Abundant) proteins in the dry seeds [34]. In our study, candidate genes *SiLEA1* and *SiLEA31* were predicted to encode LEA proteins. Interestingly, their expression levels in the caryopsis of JG21 were significantly higher than that of GBS, suggesting their potential role in regulating grain size.

Although many genes related to grain structure have been identified in other species, few have been studied in foxtail millet. Therefore, it is necessary to validate the function of these genes we identified to uncover the regulatory genes governing grain structure in foxtail millet.

## 4. Materials and Methods

### 4.1. Plant Materials

The grains were derived from a population of RILs constructed by germplasms GBS and JG21, which was previously described by Ma et al. [38]. A total of 100 lines and 2 parents were selected for this study. The plants were cultivated in the experimental field of Shanxi Agricultural University (37.43° N, 112.59° E) in 2021. The experimental site was a type of brown soil, with medium soil fertility, and the terrain was flat. The altitude of the experimental site was 802 m, the average annual temperature was 9.7 °C, the average annual precipitation was 440.7 mm, the average annual wind speed was 2.3 m/s, and the minimum daily sunshine duration was 253.5 h. Each variety was planted in 2 rows, with a row length of 2.5 m and a row spacing of 0.4 m. The seeds were sown in mid-May and harvested in early October. No fertilization or any chemical reagents were used in the field. After the grains mature, three panicles with consistent growth were chosen from the same plants. After natural drying (the water content of the dry seeds was about 14% as determined by the FOSS NIRS DS2500), the grains from the middle of the panicles were collected for Micro-CT scanning. For transcriptome sequencing, grains were taken from the middle panicle of both parents at the mid-grain filling stage and stored in liquid nitrogen.

### 4.2. Data Acquisition and Analysis

X-ray scanning of the foxtail millet grains was performed in the institute of genetics and developmental biology, Chinese academy of sciences (IGDB, CAS, Beijing, China) [39]. The scanning parameters included a spatial resolution of 3 μm, an acceleration energy of 60 kV, a current of 100 μA, and a detector exposure time of 0.3 s. Each grain underwent a 24 min scanning process, acquiring 720 frames for subsequent image reconstruction and analysis. A series of reconstructed virtual images of grain cross-section in 16-unbit BMP format were obtained using VoxelStudio Recon (Sanying Precision Instruments Co., Ltd., Tianjin, China, V2.2.3.6) software.

Traditional OpenCV image processing techniques were utilized for the segmentation and reconstruction of CT images. Based on the varying X-ray absorption of different substances, the CT images were processed using Otsu’s automatic thresholding method. By minimizing inter-class variance, the optimal threshold was determined to effectively extract the target objects in the CT images. Subsequently, a multi-stage edge detection process based on the classic Canny edge detection algorithm efficiently captured edge information in the images, providing a reliable foundation for subsequent segmentation. Following the edge detection operations, the image boundaries were selected for region growing. By aggregating adjacent pixels based on pixel similarity, regions with similar attributes were formed, and then each region in the 2D CT image was assigned a separate label and color using region growing techniques. Finally, segmentation of various portions in the images was achieved.

The AVIZO software (https://en.wikipedia.org/wiki/Avizo, accessed on 3 June 2025) was then employed to calculate various parameters, including the volume (in mm^3^) of the whole grain (K-Volume), embryo (EM-Volume), endosperm (EN-Volume), cavity (C-Volume), and hull (H-Volume); the surface area (in mm^2^) of the whole grain (K-Area), embryo (EM-Area), endosperm (EN-Area), cavity (C-Area), and hull (H-Area); and the average hull thickness (H-A-T) in millimeters. Finally, the ratios of embryo volume (EM-Ratio), endosperm volume (EN-Ratio), cavity volume (C-Ratio), and hull volume (H-Ratio) to the whole grain volume were calculated using Excel (Microsoft Office Excel 2007).

### 4.3. QTL Mapping and Transcriptome Analysis

The genotypic data [39,40] and trait data were analyzed with QTL IciMapping 4.2 software to detect QTLs [41]. The inclusion composite interval mapping (ICIM) method was used to calculate *p*-values. The QTL analysis was performed using the BIP function with Step (cM) = 1, PIN = 0.01 and permutation times were 1000 (Table S3). QTLs with LOD scores exceeding 2.5 were identified, and the confidence intervals were estimated using the 2.0 LOD-drop method. The QTLs were named using the letter ‘q’, followed by the trait abbreviation and chromosome number. Furthermore, to narrow down the range of candidate genes, we selected developing grains with similar growth characteristics from the middle portion of the two parental panicles at the middle grain filling stage for transcriptome sequencing. The cDNA library preparations and sequencing experiments were conducted by the sequencing cooperation of Novogene Co., Ltd. (Beijing, China).

### 4.4. Functional Analysis of Candidate Genes

Genes within the QTL physical interval were identified and downloaded from MDSi: Multi-omics Database for *Setaria italica* (http://sky.sxau.edu.cn/MDSi.htm, accessed on 5 September 2025). The KEGG enrichment analysis was performed using OmicShare Tools (https://www.omicshare.com, accessed on 20 September 2025).

## 5. Conclusions

Micro-CT scanning can quickly obtain three-dimensional non-destructive structural images of foxtail millet grains. By combining the differences in X-ray absorption rates of different portions in foxtail millet grains with tissue edge recognition technology, each portion can be successfully segmented. The volumes and surface areas of each portion can be calculated using AVIZO. By integrating QTL mapping and transcriptome analysis, candidate genes related to the structure of foxtail millet grains were screened out. This study has, for the first time, established a three-dimensional phenotypic analysis system for the grain structure in foxtail millet, laying the foundation for a deeper understanding of the formation of foxtail millet grain structure.

## Figures and Tables

**Table 1:** Results of statistical analysis for grain phenotypic traits.

Trait	Parents		RIL Population			CV (%)	Skewness	Kurtosis	*p*
	GBS	JG21	Minimum	Maximum	Mean ± SD				
K-Volume	2.344	3.516	2.229	3.714	3.023 ± 0.305	10.1	−0.301	0.483	0.042 *
K-Area	9.255	11.634	8.832	12.525	10.950 ± 0.735	6.71	−0.464	0.703	0.454
EM-Volume	0.200	0.359	0.178	0.361	0.259 ± 0.035	13.602	0.389	0.33	0.989
EM-Area	3.159	5.468	2.603	5.468	3.502 ± 0.475	13.555	1.271	2.981	0.000 **
EM-Ratio	0.081	0.110	0.068	0.113	0.086 ± 0.007	8.544	0.718	1.532	0.231
EN-Volume	0.840	1.940	0.840	2.241	1.759 ± 0.210	15.335	−0.123	0.301	0.557
EN-Area	7.27	12.673	7.150	12.673	9.151 ± 0.945	11.932	1.174	3.426	0.000 **
EN-Ratio	0.561	0.555	0.512	0.619	0.585 ± 0.034	10.324	−1.112	2.092	0.007 **
C-Volume	0.632	0.485	0.375	1.048	0.563 ± 0.111	19.665	1.023	3.483	0.369
C-Area	27.55	27.456	18.112	44.842	25.769 ± 3.497	13.571	1.59	7.695	0.208
C-Ratio	0.230	0.146	0.109	0.33	0.187 ± 0.035	18.739	0.845	2.676	0.168
H-Volume	0.345	0.543	0.244	0.603	0.410 ± 0.084	20.568	0.112	−0.456	0.812
H-Area	22.17	28.259	18.174	28.259	23.596 ± 1.928	8.172	0.056	0.512	0.149
H-Ratio	0.126	0.164	0.09	0.212	0.135 ± 0.022	16.724	0.319	0.811	0.908
H-A-T	35.46	43.380	26.16	56.29	38.966 ± 6.517	16.074	0.221	−0.445	0.900

Note: Asterisks indicate significant differences between the varieties by Shapiro–Wilk test (* *p* < 0.05, ** *p* < 0.01). K, the whole grain; EM, embryo; EN, endosperm; C, cavity; H, hull; H-A-T, the average thickness of the hull. The unit for volume is cubic millimeters, the unit for surface area is square millimeters, and the unit for the average thickness of the hull is micrometers.

**Table 2:** QTLs detected for six traits in the RIL population.

QTL	Chromosome	Physical Interval (Mb)	Traits	LOD	PVE (%)	Add
qKV2	2	25.180–28.330	K-Volume	3.352	17.087	0.120
			EM-Volume	3.376	15.961	0.014
			EN-Volume	3.007	14.513	0.069
			K-Area	2.663	16.719	0.262
qER3	3	12.320–14.800	EM-Ratio	2.955	11.398	0.003
qER4	4	12.960–30.600	EM-Ratio	2.818	13.170	−0.005
qHA9	9	57.300–58.220	H-Area	3.045	13.792	−0.701

**Table 3:** Candidate genes for grain structure and their homologous genes in rice.

Genes ID	Gene Names	Homologous Gene	Function Annotation of Homologous Genes
*Si2g18440*	*SiUDP82A1*	*LOC_Os04g44354*	UDP-glycosyltransferases affect cell proliferation and expansion by regulating auxin transport, thereby regulating seed development [31,32].
*Si3g18740*	*SiUDP*	*LOC_Os05g42020*	
*Si3g18760*	*SiUDP73E1*	*LOC_Os05g42040*	
*Si3g19180*	*SiWAT1*	*LOC_Os05g41420*	Regulating auxin exporting affects the elongation and division of cells [33].
*Si4g12830*	*SiLEA1*	*LOC_Os06g21910*	The mutant *mdn1-1* exhibits an increased seed size and late embryogenesis abundant (LEA) proteins are over-accumulated in the dry seeds of *mdn1-1* [34].
*Si4g14860*	*SiLEA31*	*LOC_Os06g23350*	
*Si4g17090*	*SiAP2*	*LOC*_*Os06g36000*	The mutants exhibited larger seed size and increased seed mass [35].

## Data Availability

The raw sequencing data generated in this study are available in SRA (https://www.ncbi.nlm.nih.gov/sra, accessed on 9 October 2025) of NCBI with the accession numbers PRJNA1338945.

## Supplementary Materials

The following supporting information can be downloaded at https://www.mdpi.com/article/10.3390/plants14233603/s1. Table S1: The differentially expressed genes identified in the hull between GBS and JG21. Table S2: The differentially expressed genes identified in the caryopsis between GBS and JG21. Table S3: The empirical LOD threshold (at 95% confidence) obtained from the permutation test.

## Abbreviations

The following abbreviations are used in this manuscript:

QTL	Quantitative trait loci
RILs	Recombination inbred lines
SEM	Scanning Electron Microscopy
TEM	Transmission electron microscope
CV	Coefficient of phenotypic variation
PVE	Phenotypic variation
LOD	Logarithm of odds scores
Add	Additive effects
DEGs	Differentially expressed genes
